# Insights into immune-related adverse events in colorectal cancer patients receiving neoadjuvant immunotherapy: findings from a multicenter registry study

**DOI:** 10.3389/fimmu.2025.1529637

**Published:** 2025-06-09

**Authors:** Chentong Wang, Quan Wang, Jiaolin Zhou, Aiping Zhou, Xiaojian Wu, Guanyu Yu, Lei Zhou, Yuping Zhu, Weijie Chen, Xiaoyuan Qiu, Liting Sun, Yang Gong, Xiao Zhang, Ganbin Li, Yang An, Han Chen, Xiaoyu Xie, Jinhua Tao, Guole Lin, Hongwei Yao, Wei Zhang

**Affiliations:** ^1^ Department of General Surgery, Peking Union Medical College Hospital, Peking Union Medical College, Beijing, China; ^2^ Department of Gastrocolorectal Surgery, General Surgery Center, The First Hospital of Jilin University, Changchun, Jilin, China; ^3^ Department of Medical Oncology, National Cancer Center/National Clinical Research Center for Cancer/Cancer Hospital, Chinese Academy of Medical Sciences and Peking Union Medical College, Beijing, China; ^4^ Guangdong Institute of Gastroenterology, Department of General Surgery, Guangdong Provincial Key Laboratory of Colorectal and Pelvic Floor Diseases, The Sixth Affiliated Hospital, Sun Yat-sen University, Guangzhou, Guangdong, China; ^5^ Department of Colorectal Surgery, Changhai Hospital, Naval Medical University, Shanghai, China; ^6^ Department of General Surgery, China-Japan Friendship Hospital, No.2 East Yinghua Road, Chaoyang District, Beijing, China; ^7^ Department of Colorectal Surgery, Zhejiang Cancer Hospital, 1 Banshan East Road, Hangzhou, Zhejiang, China; ^8^ Department of General Surgery, Beijing Friendship Hospital, Capital Medical University & National Clinical Research Center for Digestive Diseases, Beijing, China

**Keywords:** immune checkpoint inhibitors, colorectal neoplasms, neoadjuvant therapy, drug toxicities, immunotherapy

## Abstract

**Background:**

The growing use of immune checkpoint inhibitors (ICIs) in the neoadjuvant treatment of colorectal cancer (CRC) has highlighted immune-related adverse events (irAEs) as a major concern. This study aimed to investigate the characteristics of irAEs.

**Methods:**

This study was a retrospective, multicenter, registry-based investigation conducted in China, including 148 patients who developed irAEs after neoadjuvant immunotherapy between September 2020 and March 2024. The study analyzed the types, severity, risk factors and management strategies of irAEs. Data were collected on patient demographics, tumor assessments, neoadjuvant therapy regimens, and irAEs. Statistical analyses were conducted to identify the characteristics of irAEs and to assess their impact on surgical outcomes.

**Results:**

Among the 148 patients, a total of 203 irAEs were documented, primarily affecting the skin, endocrine system, and liver. Most irAEs (95.6%) were mild-to-moderate in severity and were effectively managed with symptomatic treatment. Hepatotoxicity was the most frequent irAE, notably associated with the combination of radiotherapy and the CAPOX chemotherapy regimen. The severity of irAEs did not affect surgical complexity or postoperative complications.

**Conclusion:**

Neoadjuvant immunotherapy combined with chemoradiotherapy demonstrates a favorable safety profile, with most irAEs being manageable. The findings support the clinical feasibility of combined regimens in CRC treatment, emphasizing the need for individualized management and extended follow-up for late-onset or chronic irAEs.

## Highlights

- Most immune side effects are mild, manageable, and don’t complicate surgery.
- Study supports tailored management of irAEs for safer CRC immunotherapy integration.
- Immune-related side effects common with neoadjuvant immunotherapy for CRC.
- Hepatotoxicity is the most frequent irAE, linked to chemoradiotherapy.

## Introduction

Colorectal cancer (CRC) is a common digestive system malignancy, ranking second in the overall incidence of cancer and fifth in overall mortality rate in China (1). Immune checkpoint inhibitors (ICIs), particularly inhibitors targeting programmed cell death protein-1 (PD-1) and cytotoxic T lymphocyte-associated protein-4 (CTLA-4), have shown promising therapeutic potential for patients with locally advanced rectal cancer (LARC) and metastatic CRC (2, 3). However, most CRC cases are microsatellite stable (MSS)/mismatch repair proficient (pMMR), limiting the effectiveness of ICIs as monotherapy. Patients undergoing neoadjuvant therapy tend to have better immune function, stronger antigen exposure, and a longer immune memory (4). Combining immunotherapy with chemotherapy or radiotherapy has emerged as a promising new strategy for CRC treatment (5).

With the application of ICIs in CRC treatment, immune-related adverse events (irAEs) have become a significant concern (6). These toxic reactions are caused by nonspecific immune system activation and can affect various organs or systems, including the skin, digestive, endocrine, and musculoskeletal systems. Common manifestations include rashes, thyroid dysfunction, and elevated liver enzymes (7). Radiotherapy and chemotherapy can enhance the effectiveness of immunotherapy by releasing tumor-associated antigens through tumor cell destruction (8). The risk factors for different types of irAEs remain unclear, particularly the role of combining immunotherapy with chemoradiotherapy. The impact of irAEs on surgical safety and postoperative complications is not yet well understood.

Existing research on irAE occurrence has largely been limited by small sample sizes, with a predominant focus on single types of adverse events rather than a comprehensive evaluation. Current insights are primarily derived from clinical trials, where irAEs are often treated as secondary outcomes and studied without a comprehensive investigation into their diversity and complexity (3, 9–15)​. As a result, a comprehensive understanding of the irAE profile in CRC neoadjuvant immunotherapy is limited, emphasizing the need for larger and more systematic evaluations.

This study therefore aims to characterize irAEs in CRC patients receiving neoadjuvant immunotherapy and to assess the safety of combining ICIs with chemoradiotherapy. By analyzing data from a large, multicenter registry cohort, this investigation will provide evidence on the safety of these combination therapies, thereby supporting future clinical decision-making and improving irAE management.

## Methods

### Study design and participants

**Figure 1:**
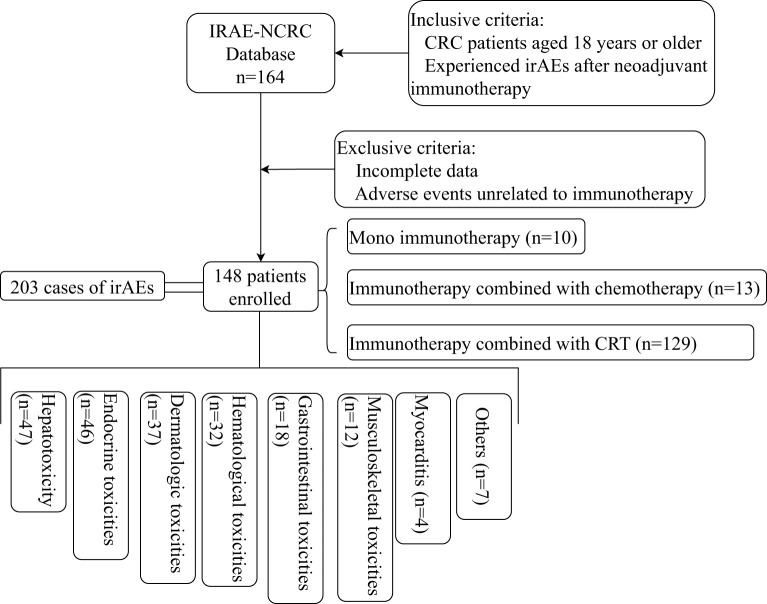
Flowchart of Patient Selection and Classification of irAEs. irAEs, immune-related adverse events; CRC, colorectal cancer.

This study was a retrospective, multicenter, registry-based investigation of CRC patients who developed irAEs during neoadjuvant immunotherapy. This cohort study involved eight tertiary referral centers across China from September 2020 to March 2024. The enrollment flowchart is shown in **Figure 1**. Eligible participants included CRC patients aged 18 years or older. All patients received ICIs as part of their treatment regimen, including PD-1 inhibitors (tislelizumab, sintilimab, toripalimab, and pembrolizumab) and the PD-1/CTLA-4 bispecific antibody cadonilimab. For analysis, patients were grouped by mechanism of ICIs (PD-1 inhibition vs. PD-1/CTLA-4 dual blockade). The diagnosis of irAEs was determined by the adverse event management teams at each participating center, while the treatment plan was decided by the physicians at each center. A database named IRAE-NCRC (irae-ncrc.com/crc/user/login) was established to consolidate data from these centers. After patient information was collected, professional physicians at the main center performed a secondary screening of the data, referencing the guidelines for managing immune checkpoint inhibitor-related toxicities, with the goal of retaining only those adverse events related to ICIs. The ethics committees of Peking Union Medical College Hospital (PUMCH) (ID: I-24PJ0024) and each participating center approved the study. Patients were monitored from the initiation of immunotherapy to at least six months post-treatment completion.

### Data collection

Data were collected and entered into the database by principal investigators at each participating center, with periodic quality checks conducted by PUMCH. The following data were collected: demographics [age at CRC diagnosis, gender, comorbidities, and body mass index (BMI)], oncological assessments (biopsy results, rectal MRI, TNM stage, expression of MMR protein, and postoperative pathology). Additionally, baseline blood test results, detailed neoadjuvant therapy regimens, and comprehensive surgical details were documented.

In this study, early-onset irAEs were defined as those occurring within three months of starting ICIs, while late-onset irAEs were defined as those occurring after three months from treatment initiation or after treatment cessation (16). Each irAE was characterized by its type, time of onset, severity, management strategy, and outcome. Adverse events were categorized and graded according to the National Cancer Institute’s Common Terminology Criteria for Adverse Events (CTCAE), version 5.0. Grades were defined as follows: Grade I (asymptomatic or mild symptoms), Grade II (moderate symptoms requiring minimal intervention), Grade III (severe symptoms), Grade IV (life-threatening symptoms), and Grade V (fatal events).

### Statistical

Descriptive statistics were employed to summarize patient demographics, baseline characteristics, and management strategies for adverse events. Categorical variables were presented as frequencies (%) and compared using the Chi-square or Fisher’s exact test. Continuous variables were analyzed based on their distribution: normally distributed data were expressed as mean ± standard deviation (SD) and analyzed via Student’s t-test, while non-normally distributed data were presented as median (Q1, Q3) and analyzed with the Mann-Whitney U test. A p-value of less than 0.05 was considered statistically significant. Indicators with a p-value less than 0.1 were included in a multivariate logistic regression analysis. Given the multiple comparisons involved in this study, a Bonferroni correction was applied to control for type I error. All statistical analyses were conducted using SPSS version 26.0 (IBM Corporation, Chicago, IL), employing modules for categorical and continuous data analysis.

## Results

### Characteristics of population and treatment regimens

**Figure 2:**
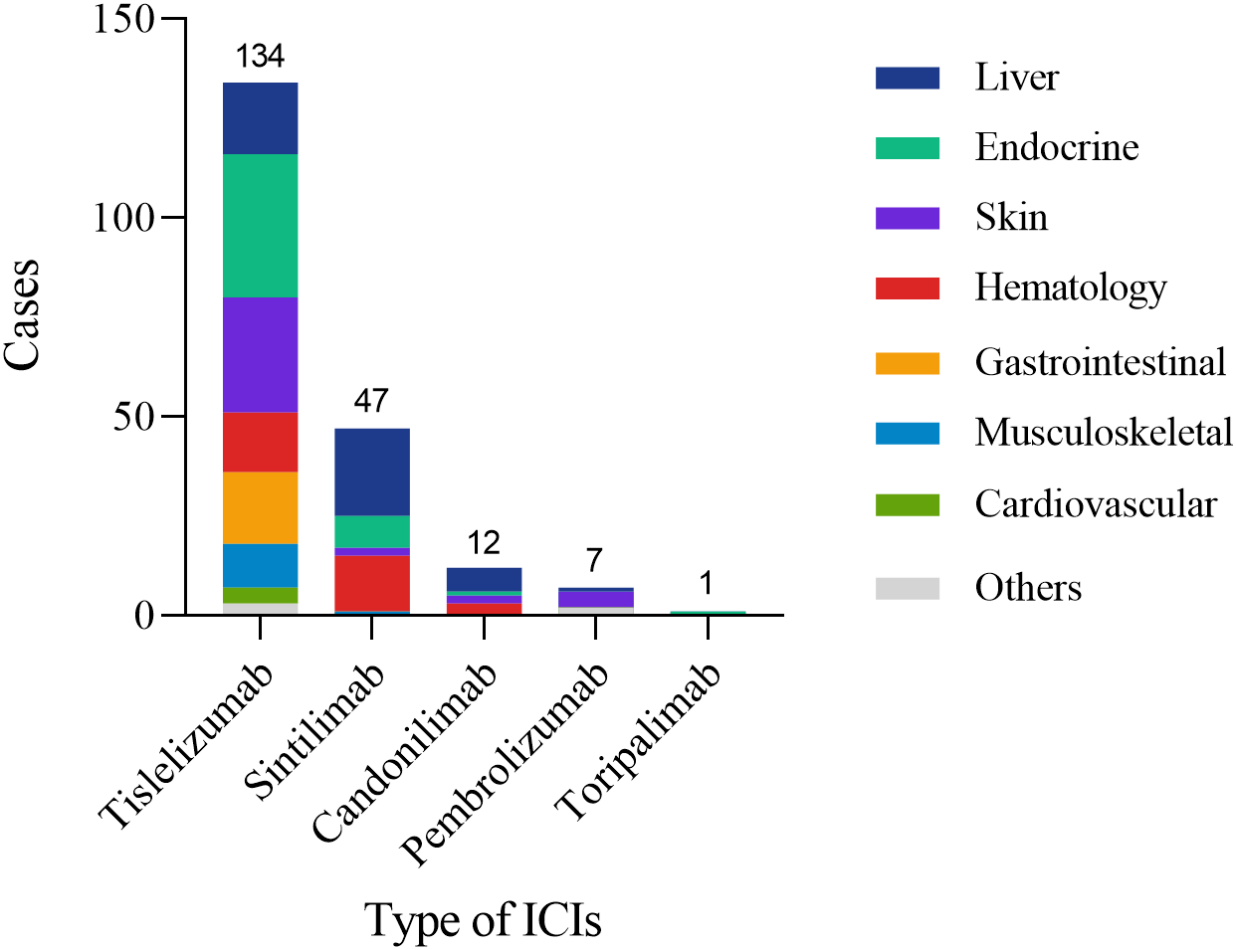
Organ distribution of irAEs among patients receiving different ICIs. The figure displays the number of patients experiencing irAEs for each ICI (Tislelizumab: 134; Sintilimab: 47; Camrelizumab: 12; Pembrolizumab: 7; Toripalimab: 1) and their distribution across affected organ systems (Liver, Endocrine, Skin, Hematology, Gastrointestinal, Musculoskeletal, Cardiovascular, Others).

This study included a cohort of 148 CRC patients who developed irAEs following treatment with ICIs. Among these patients, 94 (63.5%) were male, with a median age of 60 years (52, 67). Most patients were diagnosed with rectal cancer (93.2%) and had MSS/pMMR status (78.4%) (**Table 1**). The neoadjuvant therapy regimens administered were summarized in **Table 2**. Most patients (125, 84.5%) received a combination of ICIs and chemoradiotherapy. The primary regimen among these patients was capecitabine plus oxaliplatin (CAPOX) in combination with long-course radiotherapy (LCRT). A smaller proportion of patients (6.8%) received immunotherapy as a monotherapy. PD-1 inhibitors were the predominant ICIs used in the study cohort (93.2%). Among the different ICIs regimens, we observed distinct patterns of irAEs associated with specific agents. Tislelizumab-treated patients predominantly exhibited endocrine and cutaneous toxicities, whereas hepatic adverse events were more frequent with Sintilimab and Cadonilimab therapy, a bispecific PD-1/CTLA-4 antibody administered to 10 patients (6.8%). Pembrolizumab was primarily associated with cutaneous manifestations (**Figure 2**).

**Table 1:** Baseline characteristics and clinical outcomes stratified by irAE severity in CRC patients receiving ICIs.

Variables	Total (n = 148)	Grade I (n = 95)	Grade II-IV (n = 53)	P
Time of onset since ICIs initiation, day, M (Q_1_, Q_3_)	26.5 (19.0, 61.0)	27.0 (20.0, 60.5)	26.00 (19.0, 61.0)	0.892
Gender, Male, n(%)	94 (63.5)	61 (64.2)	33 (62.3)	0.814
Age, y, M (Q_1_, Q_3_)	60.0 (52.0, 67.0)	60.0 (52.0, 65.0)	62.0 (53.0, 69.0)	0.265
TNM stage, n(%)				0.342
I	5 (3.4)	4 (4.2)	1 (1.9)	-
II	25 (16.9)	13 (13.7)	12 (22.6)	-
III	118 (79.7)	78 (82.1)	40 (75.5)	-
Radiotherapy, n(%)				0.694
SCRT	12 (8.1)	9 (9.5)	3 (5.7)	-
LCRT	113 (76.4)	72 (75.8)	41 (77.4)	-
None	23 (15.5)	14 (14.7)	9 (17.0)	-
Chemotherapy, n(%)				0.329
Capecitabine	95 (64.2)	59 (62.1)	36 (67.9)	-
CAPOX	43 (29.1)	31 (32.6)	12 (22.6)	-
None	10 (6.8)	5 (5.3)	5 (9.4)	-
Immunotherapy, n(%)				0.460
PD-1 inhibitor	138 (93.2)	87 (91.6)	51 (96.2)	
Cadonilimab	10 (6.8)	8 (8.4)	2 (3.8)	
Time of ICI, n(%)				0.542
Concurrent plan	82 (55.4)	52 (54.7)	30 (56.6)	
Sequential plan	56 (37.8)	38 (40.0)	18 (34.0)	
Monoimmunotherapy	10 (6.8)	5 (5.3)	5 (9.4)	
Patients with pAID, yes, n(%)	21 (14.2)	11 (11.6)	10 (18.9)	0.223
HBP, yes, n(%)	23 (15.5)	13 (13.7)	10 (18.9)	0.404
DM, yes, n(%)	10 (6.8)	5 (5.3)	5 (9.4)	0.530
BMI, kg/m², Mean ± SD	23.51 ± 3.36	23.28 ± 3.18	23.91 ± 3.67	0.282
Expression of MMR proteins, n(%)				0.112
pMMR	95 (64.2)	62 (65.3)	33 (62.3)	-
dMMR	13 (8.8)	5 (5.3)	8 (15.1)	-
Not tested	40 (27.0)	28 (29.5)	12 (22.6)	-
pCR, yes, n(%)	51 (34.5)	34 (35.8)	17 (32.1)	0.798
Cycles of ICIs treatment, >3,	18 (12.2)	10 (10.5)	8 (15.1)	0.415

The table compares demographic, treatment, and comorbidity variables between patients with Grade I (n=95) and Grade II-IV (n=53) irAEs. Statistical comparisons were performed using chi-square tests for categorical variables and Mann-Whitney U tests for continuous variables. All tests were two-tailed with α=0.05. No significant differences observed in time to onset (median 26.5 days, p=0.892), demographics, or treatment parameters. dMMR showed trend toward higher-grade irAEs (15.1% vs 5.3%, p=0.112). Comorbidities and treatment outcomes did not associate with severity. SCRT, short-course radiotherapy; LCRT, long-course chemoradiotherapy; CAPOX, capecitabine plus oxaliplatin; pAID, preexisting autoimmune disease; HBP, high blood pressure; DM, diabetes mellitus; MMR, mismatch repair; Z, Mann-Whitney test; χ², Chi-square test; -, Fisher exact; M, Median; Q_1_, 1st Quartile; Q_3_, 3st Quartile.

**Table 2:** Treatment strategies in CRC patients receiving ICIs (N=148).

Treatment strategy	n (%)
Monoimmunotherapy (PD-1 inhibitor)	10 (6.8)
Immunotherapy plus chemotherapy	12 (8.1)
PD-1 inhibitor plus CAPOX	2 (1.4)
Cadonilimab plus CAPOX	10 (6.8)
Immunotherapy plus chemoradiotherapy	126 (85.1)
PD-1 inhibitor plus CAPOX and LCRT	22 (14.9)
PD-1 inhibitor plus CAP and LCRT	92 (62.2)
PD-1 inhibitor plus CAPOX and SCRT	8 (5.4)
PD-1 inhibitor plus CAP and SCRT	4 (2.7)

Data show treatment modality distribution, with the majority receiving immunotherapy plus chemoradiotherapy (85.1%). The most common regimen was PD-1 inhibitor plus capecitabine with LCRT (62.2%). Monoimmunotherapy accounted for 6.8% of cases. CAPOX, capecitabine plus oxaliplatin; SCRT, short-course radiotherapy; LCRT, long-course chemoradiotherapy; CAP, capecitabine.

### Surgical approaches and complications

Of the 148 patients, 141 (95.3%) underwent surgical procedures, with laparoscopic surgery as the most commonly employed technique (130 cases, 87.8%). Laparoscopic procedures included the Dixon procedure (64.9%), transanal total mesorectal excision (taTME) (14.9%), and the Miles procedure (2.0%). Seven patients (4.7%) opted for a non-surgical, watch-and-wait approach, mainly driven by a preference for sphincter preservation.

Postoperative complications were observed in 27 patients (19.0%) and were classified according to the Clavien–Dindo system. Among these, 23 cases were Grade I–II complications, while only four patients experienced Grade III–IV complications (**Table 3**). In terms of intraoperative metrics, Average surgery duration was 192.2 ± 59.8 minutes, and mean blood loss was 47.75 ± 23.65 ml. To assess the influence of irAEs on surgical complexity and outcomes, patients were categorized into two groups based on irAE severity: mild (Grade I) and moderate-to-severe (Grades II–IV). No statistically significant differences between these two groups in terms of surgery duration, intraoperative blood loss, or the incidence of postoperative complications were observed (**Table 3**). Pathological complete response (pCR) was achieved in 52 patients (36.6%) (**Table 3**). Additionally, 62 patients (44.0%) received adjuvant therapy, with 5 patients continuing ICIs as part of their adjuvant treatment (**Supplementary eTable 1**).

**Table 3:** Surgical outcomes stratified by irAE severity in CRC patients receiving ICIs.

Variables	Total (n = 148)	Grade I (n = 95)	Grade II-IV (n = 53)	P
Length of surgery, min, Mean ± SD	192.19 ± 59.78	196.14 ± 56.14	184.92 ± 65.93	0.287
Intraoperative blood loss, ml, Mean ± SD	47.75 ± 23.65	47.93 ± 25.27	47.40 ± 20.58	0.898
Surgical complications, yes, n (%)	27 (19.0)	16 (17.4)	11 (22.0)	0.624
Clavien-Dindo I-II	23 (15.6)	13 (13.7)	10 (18.9)	
Clavien-Dindo III-IV	4 (2.7)	3 (3.2)	1 (1.9)	

No significant differences were observed between Grade I and Grade II-IV groups for operative duration (mean 192.19 ± 59.78 vs 184.92 ± 65.93 minutes, p=0.287), blood loss (47.75 ± 23.65 vs 47.40 ± 20.58 mL, p=0.898), or overall complication rates (17.4% vs 22.0%, p=0.624). Statistical comparisons performed using independent t-tests for continuous variables and chi-square tests for categorical variables (α=0.05). SD, Standard deviation.

### Characteristic of irAEs

**Figure 3:**
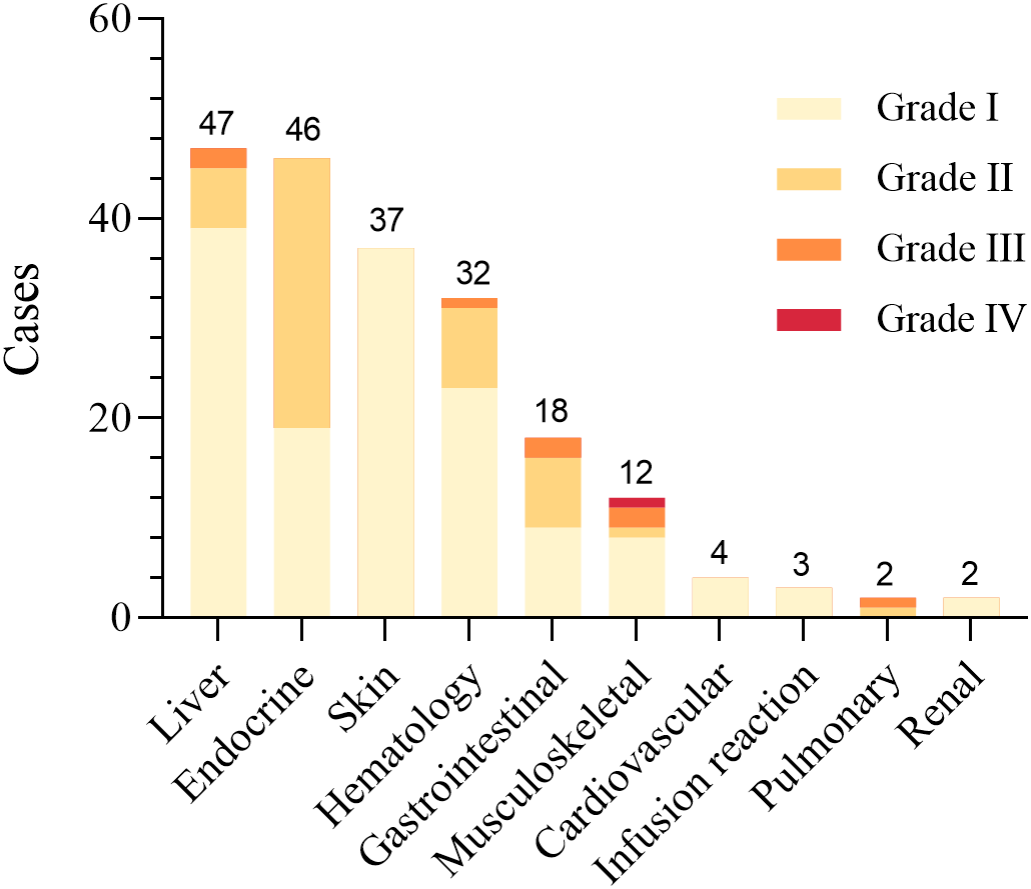
Distribution and severity grading of irAEs by organ systems in CRC patients receiving ICIs. The figure categorizes irAEs into organ systems and presents severity grading (Grade I-IV). The data demonstrate hepatic toxicity as the predominant irAE (n=47), with a subset manifesting severe clinical presentations. Endocrine toxicities (n=46) exhibited exclusively mild-to-moderate severity (Grade I-II), while dermatologic manifestations (n=37) were uniformly classified as Grade I events. Detailed results are shown in **Table 4**.

A total of 203 clinically significant irAEs were documented in this study among 148 patients receiving ICIs, affecting multiple organ systems (**Figure 3**). Among these patients, 67.6% experienced only one type of irAE, 28.4% (42 patients) had two, and 4.1% (6 patients) experienced three or more irAEs. No specific risk factors were identified for the occurrence of multiple irAEs (**Supplementary eTable 5**). Most irAEs were mild (Grade I) or moderate (Grade II), affecting various organs such as the skin, endocrine system, liver, and musculoskeletal system, and resolved with standard symptomatic treatments (**Table 4**, **Figure 3**).

**Table 4:** Spectrum, severity and onset time of irAEs in CRC patients receiving ICIs (N=148).

irAEs, n (%)	Patients (n=148)			
	Grade I	Grade II	Grade III-IV	Onset time, M (Q_1_, Q_3_)
Dermatologic toxicity	37	0	0	13 (6, 24)
Rash	25	0	0	13 (7, 25)
Pruritis	5	0	0	14 (6, 20)
RCCEP	3	0	0	16 (14.5, 33)
Bullous pemphigoid	2	0	0	1 (0.5, 1.5)
Others	2	0	0	7.5 (4.75, 10.25)
Endocrine toxicity	19	27	0	42.5 (19.25, 75)
Hypothyroidism	4	14	0	68.5 (32, 93)
Hyperthyroidism	9	2	0	61 (30.5, 72)
Diabetes mellitus	5	4	0	18 (15, 32)
Thyroiditis	0	6	0	18.5 (15.75, 22.75)
Adrenal insufficiency	1	1	0	184 (104.5, 263.5)
Hepatotoxicity	39	6	2	23 (20, 53.5)
Transaminases elevation	26	5	1	29.5 (20.75, 56.25)
Bilirubin elevation	13	1	1	20 (14, 31.5)
Musculoskeletal toxicity	8	1	3	28 (22, 32.5)
Myositis	7	0	3	28 (22.75, 31.5)
Arthritis	1	1	0	32 (27, 37)
Gastrointestinal toxicity	9	7	2	17 (12.5, 24.25)
Diarrhea	2	6	2	17.5 (11.25, 25.75)
Vomiting	7	0	0	16 (14, 22)
Colitis	0	1	0	18
Marrow toxicity	23	9	0	35 (21, 77.25)
Thrombocytopenia	15	4	0	61 (36, 92.5)
Neutropenia	1	5	0	15.5 (8.25, 22)
Anemia	6	0	0	25.5 (23, 36.25)
Eosinophilic disorders	1	0	0	23
Renal toxicity	2	0	0	92 (84.5, 99.5)
Pneumonitis	0	1	1	69.5 (68.75, 70.25)
Myocarditis	4	0	0	24.5 (20.25, 42)
Infusion reaction	2	1	0	0

The most frequent irAE was hepatotoxicity (47 cases including 2 Grade III-IV, median onset 23 days). Endocrine toxicities showed delayed presentation (median 42.5 days) with hypothyroidism being most common (18 cases). Severe (Grade III-IV) events occurred in hepatotoxicity (n=2), musculoskeletal (n=3), gastrointestinal (n=2) and pneumonitis (n=1). Onset times varied significantly by organ system, with endocrine toxicities demonstrating the longest median latency (42.5 days) and dermatologic events the shortest (13 days). RCCEP, Reactive cutaneous capillary endothelial proliferation; M, Median; Q_1_, 1st Quartile; Q_3_, 3st Quartile.

Hepatotoxicity, primarily Grade I-II reactions, was the most common irAE, affecting 47 patients (23.2%) with a median onset of 23 days (20, 53.5). 25 patients experienced spontaneous resolution without treatment, and 21 improved with hepatoprotective drugs. One patient developed Grade III transaminase elevation, resolving after multidisciplinary treatment with corticosteroids and immunosuppressants. A higher incidence of hepatotoxicity was observed in patients receiving short-course radiotherapy (SCRT) or the CAPOX chemotherapy regimen, whereas a history of hepatitis or number of immunotherapy cycles was not a risk factor (**Supplementary eTable 2**). Multivariate analysis showed that combined SCRT was an independent risk factor for hepatic toxicity [p=0.015, OR (95% CI) = 7.92 (1.50 ~ 41.84), Ref: LCRT].

Endocrine irAEs were the second most common, affecting 46 patients (22.7%) with a median onset of 42.5 days (19.25–75), which was significantly later than other irAEs. The most prevalent endocrine irAEs were hypothyroidism (18 cases), hyperthyroidism (11 cases), and thyroiditis (6 cases), often detected as asymptomatic thyroid function abnormalities in routine lab tests. Patients with thyroiditis often progressed from hyperthyroidism to hypothyroidism, requiring hormone replacement. All thyroid dysfunction cases were Grade I-II and did not require treatment modification. In addition, 9 patients developed elevated blood glucose levels, with 5 resolving through lifestyle modifications and 4 requiring oral hypoglycemic medications, though no serious complications like diabetic ketoacidosis occurred. Patients with a history of autoimmune diseases, particularly Hashimoto’s thyroiditis, were at increased risk for endocrine irAEs (**Supplementary eTable 3**).

Dermatologic toxicities were observed in 35 patients (17.2%), with a median onset of 13 days (6, 24), earlier than most other adverse reactions. Common presentations included rash (25 cases) and pruritus (5 cases), primarily categorized as Grade I, requiring only topical creams or antihistamines for relief. More severe cases, such as bullous pemphigoid (2 cases), led to treatment delays until symptoms were managed. Reactive cutaneous capillary endothelial proliferation (RCCEP), observed exclusively in pembrolizumab-treated patients, resolved spontaneously without intervention. No risk factors were found for skin toxicity (**Supplementary eTable 4**).

Musculoskeletal irAEs, such as asymptomatic creatine kinase elevations or mild myositis, were observed in 12 patients (5.9%) with a median onset time of 28 (22, 32.5) days. While most cases were mild, three severe instances required high-dose corticosteroids and immunosuppressive therapy due to symptoms like muscle weakness and dysphagia, leading to permanently discontinuation of ICIs. 1 patient with pre-existing osteoarthritis experienced worsening joint pain, managed with corticosteroids.

Additionally, there were some rare but severe adverse events, including cardiac and pulmonary toxicity. In this study, four patients developed mild cardiac toxicity, manifested by elevated myocardial enzymes and slight changes in electrocardiogram, but without obvious clinical symptoms. After discontinuing ICIs, these values gradually returned to normal, and no further treatment was affected. Two other patients experienced pulmonary toxicity, with mild dyspnea and cough, which improved following treatment with corticosteroids and immunoglobulin.

### Late-onset irAEs

</u>In total, 46 patients experienced late-onset irAEs, with a median onset of 78 days (range: 63.5–94.0), primarily affecting the endocrine and hepatic systems. The latest recorded case of a late-onset irAE was adrenal insufficiency, observed 343 days after the initiation of immunotherapy. These late-onset irAEs were significantly associated with SCRT and CAPOX therapy combinations (**Supplementary eTable 6**). However, further multivariate analysis did not identify those as independent risk factors for late-onset irAEs.

## Discussion

This study collected clinical information on CRC patients who experienced irAEs during neoadjuvant immunotherapy combined with chemoradiotherapy. Our aim was to analyze the characteristics of these irAEs. To date, this is the largest retrospective clinical study on irAEs associated with neoadjuvant immunotherapy for CRC. Our findings reveal that while irAEs are common in combined treatments, they are manageable and do not increase postoperative complications significantly.

In our study, hepatotoxicity was the most frequent irAE, contrasting with previous studies where dermatologic and endocrine toxicities are predominant in immunotherapy (17). The hepatotoxicity observed in our study, frequently manifesting as asymptomatic transaminase elevations, may be attributed either to direct hepatic injury caused by chemoradiotherapy or to synergistic immune activation resulting from its combination with immunotherapy (18, 19). Compared with LCRT, SCRT regimens may elevate hepatotoxicity risk, possibly through enhanced anti-tumor immune activation (20). For high-risk patients, adjusting the treatment regimen, such as using LCRT, may be advisable. Our study found that patients with autoimmune diseases did not have a higher risk of high-grade irAEs, suggesting that such conditions may not contraindicate immunotherapy. However, both our findings and those of Quan et al. indicate that pre-existing autoimmune diseases may be reactivated or exacerbated by ICIs (21). Thus, enhanced monitoring and management are essential for patients with a history of autoimmune diseases. Our study also found that dermatologic irAEs occurred earlier than other irAEs, while endocrine irAEs had a later onset. This suggests that the timing of irAEs varies greatly across different target organs, which may help us further understand the mechanisms underlying their occurrence.

Despite being PD-1 inhibitors, different ICIs exhibited distinct irAE patterns. Consistent with findings from the TORCH study (22), our data demonstrated a significantly higher incidence of hepatotoxicity with sintilimab treatment, potentially attributable to its frequent combination with SCRT regimens. In contrast, pembrolizumab was predominantly linked to dermatologic toxicities, likely attributable to its characteristic induction of RCCEP. Combining two ICIs targeting different pathways may create a synergistic effect, increasing the severity and range of adverse reactions (23, 24). In our study, a portion of patients were treated with Cadonilimab; however, there was no evidence to suggest that the severity of irAEs was increased in this group. Studies suggest that administering ICIs after radiotherapy may enhance immune response by promoting antigen release from tumor cell destruction (25). However, our study found no significant difference in irAE severity between concurrent and sequential administration of ICIs with chemoradiotherapy, suggesting that both approaches are feasible.

Although some researchers have suggested that ICIs may activate the immune system, potentially leading to enhanced inflammatory responses in surrounding tissues, which could obscure tissue planes and increase surgical difficulty, as well as impair wound healing (26), our study did not find that severe irAEs significantly impact operative time or the incidence of postoperative complications. On the contrary, in our study, the overall incidence of surgery-related complications across all grades in patients who experienced irAEs was lower than the rates reported in other studies for patients receiving neoadjuvant therapy (13, 27). This could be attributed to the relatively low frequency of ICI treatment during neoadjuvant therapy, leading to a milder inflammatory response. Additionally, neoadjuvant therapy significantly reduces tumor size, which may contribute to lowering surgical difficulty. Another concern is that the occurrence of irAEs may potentially delay or even prevent surgery in these patients (28). However, in our cohort, even patients with grade II or higher irAEs did not experience delays or discontinuation of surgery.

The onset of irAEs usually occurs later than chemotherapy and radiotherapy adverse reactions and can arise at any stage of treatment, with the potential for recurrence (29, 30). In our study, 46 patients experienced late-onset irAEs, primarily endocrine toxicities, which is consistent with findings from other studies (31). Some studies have found that ICI combination therapy increases the incidence of late-onset irAEs (32); however, our study did not identify any predictive factors.

Our analysis showed that for most Grade I-II irAEs, symptomatic treatment alone was effective, avoiding delays or cessation of immunotherapy. For Grade III or higher reactions, immunotherapy was paused and managed with corticosteroids or immunosuppressants, yielding favorable outcomes with this severity-based approach. All patients with Grade IV adverse reactions permanently discontinued ICIs. For other patients, ICIs were restarted once adverse reactions subsided to Grade I, with a reassessment of treatment benefits.

In summary, this study characterized the types, severity, and management of irAEs, confirming the safety of ICIs in CRC neoadjuvant treatment. These results will help optimize personalized treatment for CRC by identifying high-risk populations more likely to experience specific irAEs. Patients receiving concurrent chemoradiotherapy may be at higher risk for hepatotoxicity, while those with autoimmune diseases may require closer monitoring. Future research should focus on elucidating the mechanisms of irAEs and identifying predictive biomarkers. Long-term, prospective, and multicenter studies with diverse patient populations, especially older patients and those with comorbidities, are needed to assess the impact of irAE management on survival, quality of life, and the long-term incidence of irAEs.

### Limitations

This study has several limitations, primarily due to its retrospective, registry-based design, which may introduce selection and information biases. Additionally, the relatively young and healthy profile of our cohort may limit representativeness for older or comorbid populations, and the short follow-up period may have restricted the detection of late-onset and chronic irAEs.

## Conclusion

This study demonstrates the safety and feasibility of combining ICIs with chemoradiotherapy as neoadjuvant treatment for CRC. Most irAEs are manageable, mild-to-moderate in severity, and do not increase postoperative complications with appropriate symptomatic management. Our findings support the development of tailored management plans for CRC patients receiving ICIs and chemoradiotherapy and underscore the importance of continuous monitoring and proactive management of irAEs, particularly for high-risk groups. However, large-scale prospective studies remain necessary to further clarify irAE risks across diverse populations and to optimize personalized treatment strategies.

## Data Availability

The original contributions presented in the study are included in the article/**Supplementary Material**. Further inquiries can be directed to the corresponding author/s.
