# Supplementary material for: Insights into immune-related adverse events in colorectal cancer patients receiving neoadjuvant immunotherapy: findings from a multicenter registry study
Source: Front Immunol. 2025 Jun 9;16:1529637. doi: 10.3389/fimmu.2025.1529637 (PMC12183158; doi:10.3389/fimmu.2025.1529637)
Supplement: Supplementary file 1 [file Table1.docx]

Supplement eTable 1 Surgical approaches and postoperative outcomes in CRC patients receiving ICIs (N=148).

| Variables | n (%) |
| --- | --- |
|  |  |
| Surgical procedures | 141 (95.3) |
| Laparoscopic surgery | 130 (87.8) |
| Dixon | 96 (64.9) |
| taTME | 22 (14.9) |
| Miles | 3 (2.0) |
| Colectomy | 6 (4.0) |
| Others | 3 (2.0) |
| Robotic surgery | 11 (7.4) |
| Clavien–Dindo classification of complications |  |
| I | 10 (6.8) |
| II | 13 (8.8) |
| III-IV | 4 (2.8) |
| Adjuvant therapy | 62 (41.9) |
| Capecitabine | 18 (12.2) |
| Capecitabine plus oxaliplatin | 39 (26.4) |
| Immunotherapy plus chemotherapy | 3 (2.0) |
| Immunotherapy alone | 2 (1.4) |

The majority underwent laparoscopic procedures (87.8%), predominantly Dixon resection (64.9%). Robotic surgery accounted for 7.4% of cases. Postoperative complications occurred in 18.4% of patients, with Clavien-Dindo grade I-II complications being most frequent (15.6%). Adjuvant therapy was administered to 41.9% of patients, primarily capecitabine-based regimens (38.6%). Data presented as frequency (percentage). taTME: transanal total mesorectal excision

Supplement eTable 2 Risk Factors for Immune-Related Hepatotoxicity (n=101).

| Variables | No hepatoxicity  (n = 101) | Hepatoxicity  (n = 47) | P |
| --- | --- | --- | --- |
|  |  |  |  |
| Time of onset since ICIs initiation, day, M (Q₁, Q₃) | 26.0 (18.0, 67.0) | 23.0 (20.0, 53.5) | 0.913 |
| Gender, Male, n(%) | 65 (64.4) | 29 (61.7) | 0.755 |
| Age, y, M (Q₁, Q₃) | 61.0 (53.0, 68.0) | 55.0 (51.5, 65.0) | 0.089 |
| BMI, kg/m^2^, Mean ± SD | 23.53 ± 3.02 | 23.68 ± 3.53 | 0.783 |
| NLR, Mean ± SD | 2.54 ± 2.76 | 2.78 ± 2.63 | 0.606 |
| Radiotherapy, n(%) |  |  | <.001 |
| SCRT | 2 (2.0) | 10 (21.3) |  |
| LCRT | 82 (81.2) | 31 (66.0) |  |
| None | 17 (16.8) | 6 (12.8) |  |
| Chemotherapy, n(%) |  |  | <.001 |
| None | 10 (9.9) | 0 |  |
| CAP | 73 (72.3) | 22 (46.8) |  |
| CAPOX | 18 (17.8) | 25 (53.2) |  |
| Immunotherapy, n(%) |  |  | 0.102 |
| PD-1 inhibitor | 97 (96.0) | 41 (87.2) |  |
| Cadonilimab | 4 (4.0) | 6 (12.8) |  |
| Patients with liver disease, yes, n(%) | 2 (2.0) | 2 (4.3) | 0.802 |
| pCR, yes, n(%) | 36 (35.6) | 15 (31.9) | 0.534 |
| Cycles of ICIs treatment, ＞3, | 10 (9.9) | 8 (17.0) | 0.217 |

Patients developing hepatotoxicity were younger (median 55 vs 61 years, p=0.089) and showed significant associations with radiotherapy type (SCRT 21.3% vs 2.0%, p<0.001) and chemotherapy regimen (CAPOX 53.2% vs 17.8%, p<0.001). No significant differences were observed in baseline liver disease (4.3% vs 2.0%, p=0.802), gender distribution (61.7% vs 64.4% male, p=0.755), or pCR rates (31.9% vs 35.6%, p=0.534). The cadonilimab group showed higher hepatotoxicity incidence (12.8% vs 4.0%, p=0.102). Continuous variables analyzed by Mann-Whitney U test, categorical by chi-square/Fisher's exact test (α=0.05). NLR: neutrophil-to-lymphocyte ratio; BMI: body mass index; SCRT: short-course radiotherapy; LCRT: long-course chemoradiotherapy; CAPOX: capecitabine plus oxaliplatin; CAP: capecitabine; pCR: pathological complete response, M: Median, Q₁: 1st Quartile, Q₃: 3st Quartile, SD: Standard deviation

Supplement eTable 3 Risk Factors for Endocrine Toxicity (n=46).

| Variables | No endocrine toxicity  (n = 102) | Endocrine toxicity  (n = 46) | P |
| --- | --- | --- | --- |
|  |  |  |  |
| Time of onset since ICIs initiation, day, M (Q₁, Q₃) | 23.0 (17.25, 49.75) | 49.0 (22.0, 81.25) | <.001 |
| Gender, Male, n(%) | 66 (64.7) | 28 (60.9) | 0.654 |
| Age, y, M (Q₁, Q₃) | 60.0 (52.0, 65.0) | 62.0 (53.0, 69.0) | 0.265 |
| BMI, kg/m^2^, Mean ± SD | 23.60 ± 3.25 | 23.53 ± 3.07 | 0.915 |
| NLR, Mean ± SD | 2.74 ± 3.16 | 2.33 ± 1.25 | 0.399 |
| TNM stage, n(%) |  |  | 0.650 |
| I | 4 (3.9) | 1 (2.2) |  |
| II | 19 (18.6) | 6 (13.0) |  |
| III | 79 (77.5) | 39 (84.8) |  |
| Radiotherapy, n(%) |  |  | 0.412 |
| SCRT | 10 (9.8) | 2 (4.4) |  |
| LCRT | 75 (73.5) | 38 (82.6) |  |
| None | 17 (16.7) | 6 (13.0) |  |
| Treatment regimes, n(%) |  |  | 0.606 |
| Immunotherapy alone | 7 (6.9) | 3 (6.5) |  |
| ICIs plus chemotherapy | 10 (9.8) | 2 (4.4) |  |
| ICIs plus chemoradiotherapy | 85 (83.3) | 41 (89.1) |  |
| Immunotherapy, n(%) |  |  | 0.255 |
| PD-1 inhibitor | 93 (91.2) | 45 (97.8) |  |
| Cadonilimab | 9 (8.8) | 1 (2.2) |  |
| Patients with pAID, yes, n(%) | 9 (8.8) | 12 (26.1) | 0.005 |
| DM, yes, n(%) | 7 (6.9) | 3 (6.5) | 1.000 |
| pCR, yes, n(%) | 33 (32.4) | 18 (39.1) | 0.556 |
| Cycles of ICIs treatment, ＞3, | 15 (14.7) | 3 (6.5) | 0.159 |

Patients with endocrine toxicity demonstrated significantly delayed onset (median 49 vs 23 days, p<0.001) and higher prevalence of pre-existing autoimmune disease (pAID, 26.1% vs 8.8%, p=0.005). No significant differences were observed in baseline characteristics including age (median 62 vs 60 years, p=0.265), BMI (23.53 vs 23.60 kg/m², p=0.915), or treatment regimens (p=0.606). Statistical analysis performed using Mann-Whitney U test for continuous variables and chi-square/Fisher's exact test for categorical variables (α=0.05). NLR: Neutrophil-to-lymphocyte ratio; BMI: Body mass index; SCRT: short-course radiotherapy; LCRT: long-course chemoradiotherapy; pAID: preexisting autoimmune disease; DM: diabetes mellitus; pCR: pathological complete response, M: Median, Q₁: 1st Quartile, Q₃: 3st Quartile, SD: Standard deviation

Supplement eTable 4 Risk Factors for Skin Toxicity (n=35).

| Variables | Non skin toxicity  (n = 113) | skin toxicity  (n = 35) | P |
| --- | --- | --- | --- |
|  |  |  |  |
| Time of onset since ICIs initiation, day, M (Q₁, Q₃) | 32.0 (20.0, 68.0) | 18.0 (8.5, 27.0) | <.001 |
| Gender, Male, n(%) | 73 (64.6) | 21 (60.0) | 0.621 |
| Age, y, M (Q₁, Q₃) | 59.0 (52.0, 66.0) | 62.0 (53.0, 69.0) | 0.267 |
| BMI, kg/m^2^, Mean ± SD | 23.52 ± 3.04 | 23.76 ± 3.65 | 0.703 |
| NLR, Mean ± SD | 2.68 ± 3.02 | \| 2.39 ± 1.31 \| \| --- \| | 0.580 |
| Radiotherapy, n(%) |  |  | 0.275 |
| SCRT | 11 (9.7) | 1 (2.9) |  |
| LCRT | 83 (73.5) | 30 (85.7) |  |
| None | 19 (16.8) | 4 (11.4) |  |
| Treatment regimes, n(%) |  |  | 0.919 |
| Immunotherapy alone | 95 (84.1) | 31 (88.6) |  |
| ICIs plus chemotherapy | 10 (8.9) | 2 (5.7) |  |
| ICIs plus chemoradiotherapy | 8 (7.1) | 2 (5.7) |  |
| Immunotherapy, n(%) |  |  | 1.000 |
| PD-1 inhibitor | 105 (92.9) | 33 (94.3) |  |
| Cadonilimab | 8 (7.1) | 2 (5.7) |  |
| pCR, yes, n(%) | 38 (33.6) | 13 (37.1) | 0.865 |
| Cycles of ICIs treatment, ＞3, | 15 (13.3) | 3 (8.6) | 0.654 |

Cutaneous irAEs demonstrated significantly earlier onset (median 18 vs 32 days, p<0.001). No significant differences were observed in baseline characteristics including gender distribution (60.0% vs 64.6% male, p=0.621), age (median 62 vs 59 years, p=0.267), or BMI (23.76 vs 23.52 kg/m², p=0.703). Pathological complete response rates were similar between groups (37.1% vs 33.6%, p=0.865). Statistical analysis performed using Mann-Whitney U test for continuous variables and chi-square/Fisher's exact test for categorical variables (α=0.05). NLR: neutrophil-to-lymphocyte ratio; BMI: body mass index; SCRT: short-course radiotherapy; LCRT: long-course chemoradiotherapy; pCR: pathological complete response, M: Median, Q₁: 1st Quartile, Q₃: 3st Quartile, SD: Standard deviation

Supplement eTable 5 Risk Factors for Developing Multiple irAEs (n=48).

| Variables | Single irAE  (n = 100) | Multiple irAEs  (n = 48) | P |
| --- | --- | --- | --- |
|  |  |  |  |
| Time of onset since ICIs initiation, day, M (Q₁, Q₃) | 27.00 (19.00, 65.25) | 25.00 (19.75, 49.75) | 0.558 |
| Gender, Male, n(%) | 67 (67.0) | 27 (56.3) | 0.203 |
| Age, y, M (Q₁, Q₃) | 60.50 (51.75, 68.25) | 58.50 (52.75, 65.00) | 0.280 |
| BMI, kg/m^2^, Mean ± SD | 23.44 ± 3.26 | 23.64 ± 3.60 | 0.734 |
| NLR, Mean ± SD | 2.67 ± 2.97 | 2.50 ± 2.09 | 0.727 |
| Radiotherapy, n(%) |  |  | 0.126 |
| SCRT | 6 (6.0) | 6 (12.5) |  |
| LCRT | 75 (75.0) | 38 (79.2) |  |
| None | 19 (19.0) | 4 (8.3) |  |
| Chemotherapy, n(%) |  |  | 0.679 |
| Capecitabine | 63 (63.0) | 32 (66.7) |  |
| CAPOX | 29 (29.0) | 14 (29.2) |  |
| None | 8 (8.0) | 2 (4.2) |  |
| Immunotherapy, n(%) |  |  | 0.102 |
| PD-1 inhibitor | 97 (96.0) | 41 (87.2) |  |
| Cadonilimab | 4 (4.0) | 6 (12.8) |  |
| Treatment regimes, n(%) |  |  | 0.375 |
| Immunotherapy alone | 82 (82.0) | 44 (91.7) |  |
| ICIs plus chemotherapy | 10 (10.0) | 2 (4.2) |  |
| ICIs plus chemoradiotherapy | 8 (8.0) | 2 (4.2) |  |
| Patients with pAID, yes, n(%) | 2 (2.0) | 2 (4.3) | 0.362 |
| Cycles of ICIs treatment, ＞3, | 14 (14.0) | 4 (8.3) | 0.323 |

No significant differences were observed in time to onset (median 27 vs 25 days, p=0.558), demographic factors, or treatment parameters between groups. The cadonilimab group showed a non-significant trend toward higher multiple irAE incidence (12.8% vs 4.0%, p=0.102). Treatment regimens and baseline characteristics were comparable between groups (all p>0.05). Statistical comparisons performed using Mann-Whitney U test for continuous variables and chi-square/Fisher's exact test for categorical variables (α=0.05). NLR: Neutrophil-to-lymphocyte ratio; BMI: Body mass index; SCRT: short-course radiotherapy; LCRT: long-course chemoradiotherapy; CAPOX: capecitabine plus oxaliplatin; pCR: pathological complete response; pAID: preexisting autoimmune disease, M: Median, Q₁: 1st Quartile, Q₃: 3st Quartile, SD: Standard deviation

Supplement eTable 6 Comparative analysis of early-onset (n=102) versus late-onset (n=46) irAEs

| Variables | Early-onset irAEs (n = 102) | late-onset irAEs (n = 46) | P |
| --- | --- | --- | --- |
|  |  |  |  |
| BMI, kg/m², Mean ± SD | 23.82 ± 3.30 | 22.82 ± 3.43 | 0.093 |
| NLR, Mean ± SD | 2.57 ± 2.95 | 2.71 ± 2.12 | 0.785 |
| Gender, Male, n(%) | 68 (66.7) | 26 (56.5) | 0.235 |
| Age, y, M (Q₁, Q₃) | 61.0 (52.0, 68.0) | 59.5 (52.0, 65.0) | 0.496 |
| TNM stage, n(%) |  |  | 0.237 |
| I-II | 18 (17.7) | 12 (26.1) |  |
| III | 84 (82.4) | 34 (73.9) |  |
| Radiotherapy, n(%) |  |  | 0.038 |
| SCRT | 6 (5.9) | 6 (13.0) |  |
| LCRT | 84 (82.4) | 29 (63.0) |  |
| None | 12 (11.8) | 11 (23.9) |  |
| Chemotherapy, n(%) |  |  | 0.006 |
| Capecitabine | 74 (72.6) | 21 (45.7) |  |
| CAPOX | 22 (21.6) | 21 (45.7) |  |
| None | 6 (5.9) | 4 (8.7) |  |
| Immunotherapy, n(%) |  |  | 0.460 |
| PD-1 inhibitor | 87 (91.6) | 51 (96.2) |  |
| Cadonilimab | 8 (8.4) | 2 (3.8) |  |
| Time of ICI, n(%) |  |  | 0.138 |
| Concurrent plan | 52 (51.0) | 30 (65.2) |  |
| Sequential plan | 44 (43.1) | 12 (26.1) |  |
| Monoimmunotherapy | 6 (5.9) | 4 (8.7) |  |
| Treatment regimes, n(%) |  |  | 0.217 |
| Immunotherapy alone | 90 (88.2) | 36 (78.3) |  |
| ICIs plus chemotherapy | 6 (5.9) | 6 (13.0) |  |
| ICIs plus chemoradiotherapy | 6 (5.9) | 4 (8.7) |  |
| Patients with pAID, yes, n(%) | 15 (14.7) | 6 (13.0) | 0.789 |
| DM, yes, n(%) | 8 (7.8) | 2 (4.4) | 0.667 |
| Grades, n(%) |  |  | 0.585 |
| I | 64 (62.8) | 31 (67.4) |  |
| II-IV | 38 (37.3) | 15 (32.6) |  |
| Cycles of ICIs treatment, ＞3, n(%) | 12 (11.8) | 6 (13.0) | 0.826 |

Significant differences were observed in treatment modalities, with SCRT associated with late-onset irAEs (13.0% vs 5.9%, p=0.038) and CAPOX chemotherapy more frequent in late-onset cases (45.7% vs 21.6%, p=0.006). Early-onset irAEs showed non-significant trends toward higher BMI (23.82 vs 22.82 kg/m², p=0.093) and male predominance (66.7% vs 56.5%, p=0.235). Statistical analysis performed using independent t-tests for continuous variables and chi-square tests for categorical variables (α=0.05). NLR: Neutrophil-to-lymphocyte ratio; BMI: Body mass index; SCRT: short-course radiotherapy; LCRT: long-course chemoradiotherapy; CAPOX: capecitabine plus oxaliplatin; DM: diabetes mellitus; pCR: pathological complete response; pAID: preexisting autoimmune disease, M: Median, Q₁: 1st Quartile, Q₃: 3st Quartile, SD: Standard deviation

Supplement eTable 6. Multivariate logistic regression analysis of factors associated with late-onset irAEs in CRC patients receiving ICIs.

| Variables | β | S.E | P | OR (95%CI) |
| --- | --- | --- | --- | --- |
| BMI, kg/m², Mean ± SD | -0.05 | 0.06 | 0.399 | 0.95 (0.85 ~ 1.07) |
| Radiotherapy, n(%) |  |  |  |  |
| SCRT | Ref | Ref | Ref | Ref |
| LCRT | -0.58 | 0.67 | 0.914 | 0.91 (0.18 ~ 4.69) |
| None | -0.09 | 0.83 | 0.388 | 0.56 (0.15 ~ 2.08) |
| Chemotherapy, n(%) | 0.006 |  |  |  |
| None | Ref | Ref | Ref | Ref |
| Capecitabine | -0.17 | 1.02 | 0.867 | 0.84 (0.11 ~ 6.25) |
| CAPOX | 0.74 | 0.88 | 0.402 | 2.09 (0.37 ~ 11.80 |

BMI showed a non-significant inverse relationship (OR 0.95, 95% CI 0.85-1.07, p=0.399). Radiotherapy modalities (LCRT vs SCRT: OR 0.91, 95% CI 0.18-4.69, p=0.914; none vs SCRT: OR 0.56, 95% CI 0.15-2.08, p=0.388) and chemotherapy regimens (CAPOX vs none: OR 2.09, 95% CI 0.37-11.80, p=0.402) did not demonstrate significant predictive value. The model was adjusted for all listed covariates. Statistical significance was set at α=0.05. SCRT: short-course radiotherapy; LCRT: long-course chemoradiotherapy; CAPOX: capecitabine plus oxaliplatin, SD: Standard deviation; OR: odds ratio; S.E: standard error
